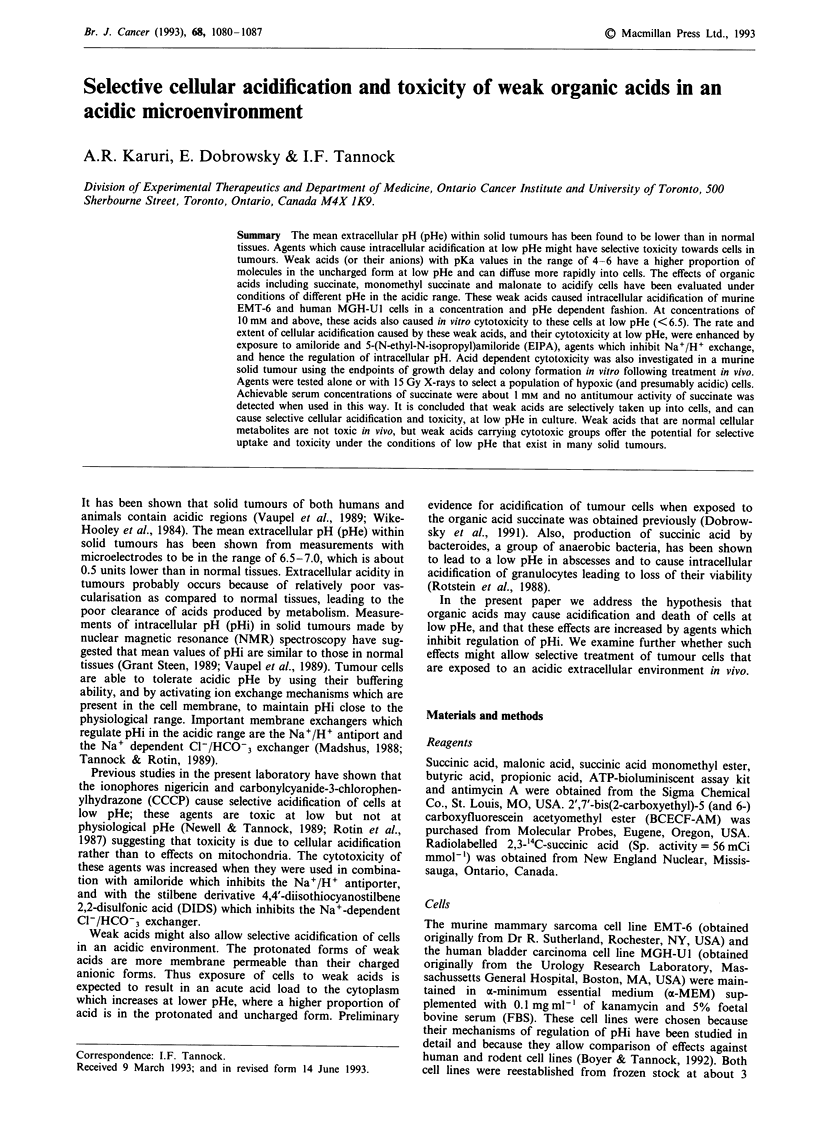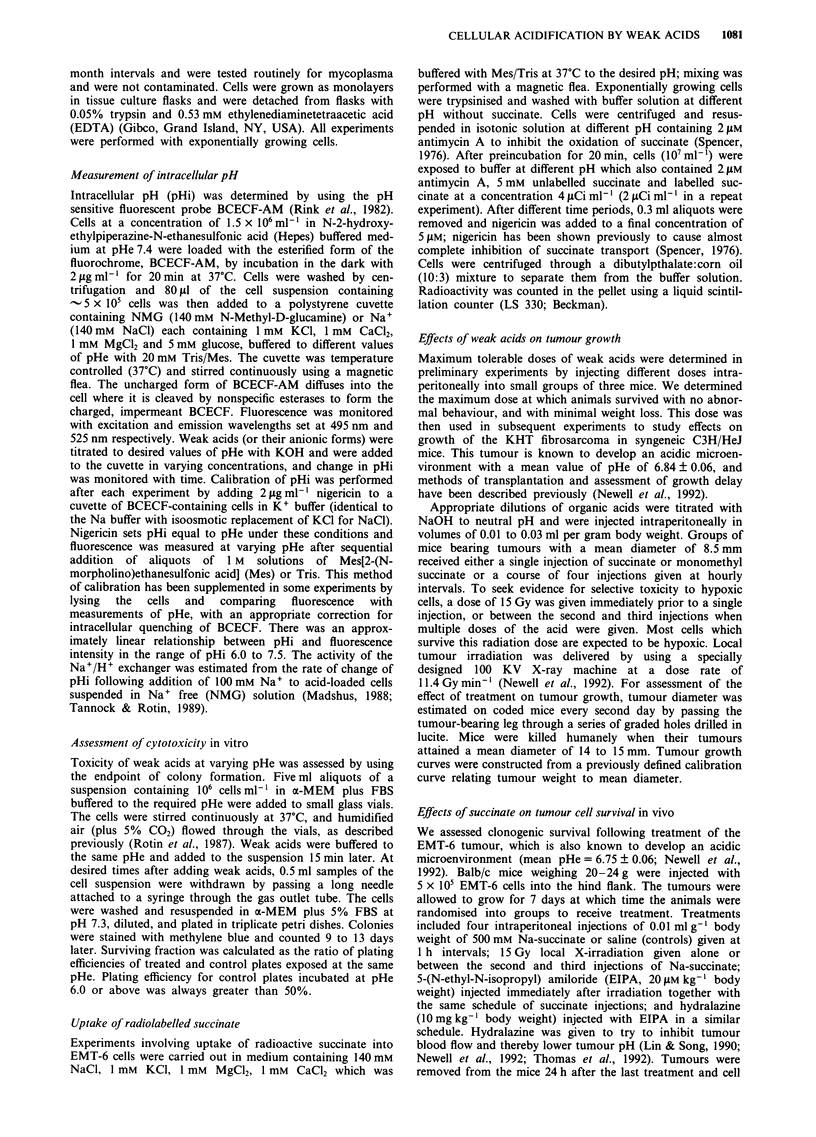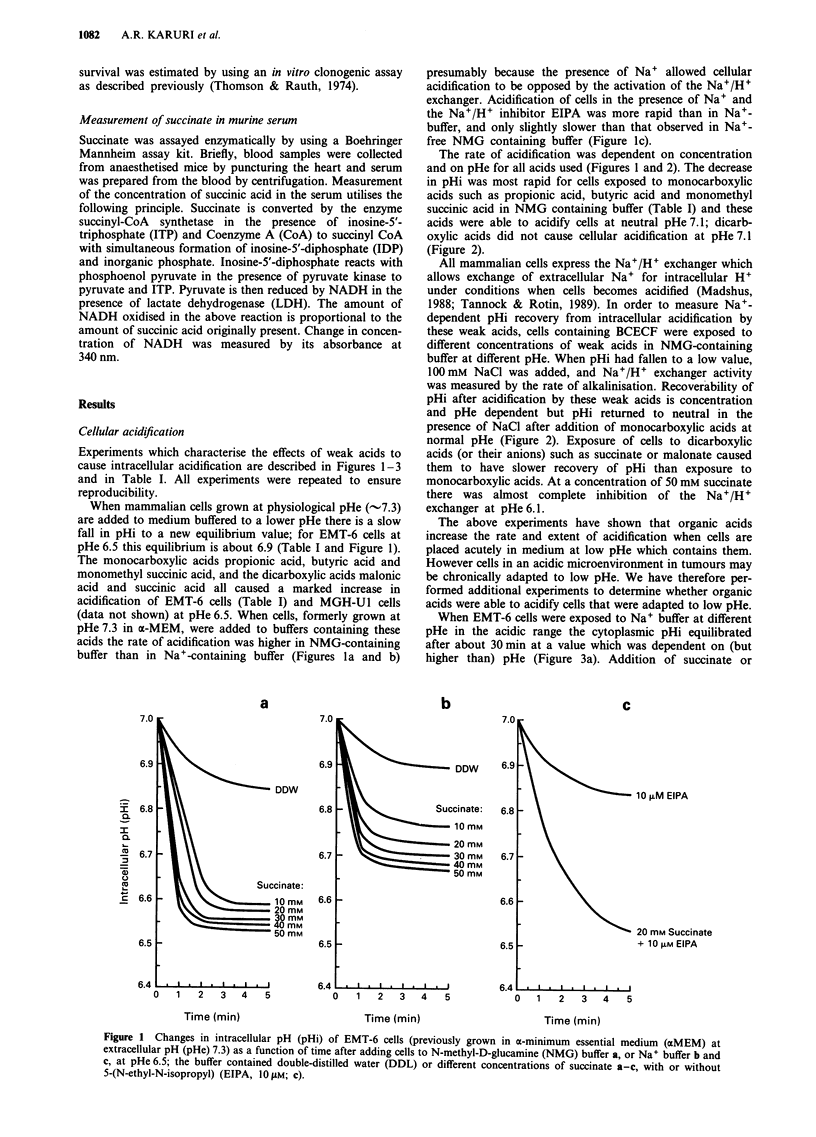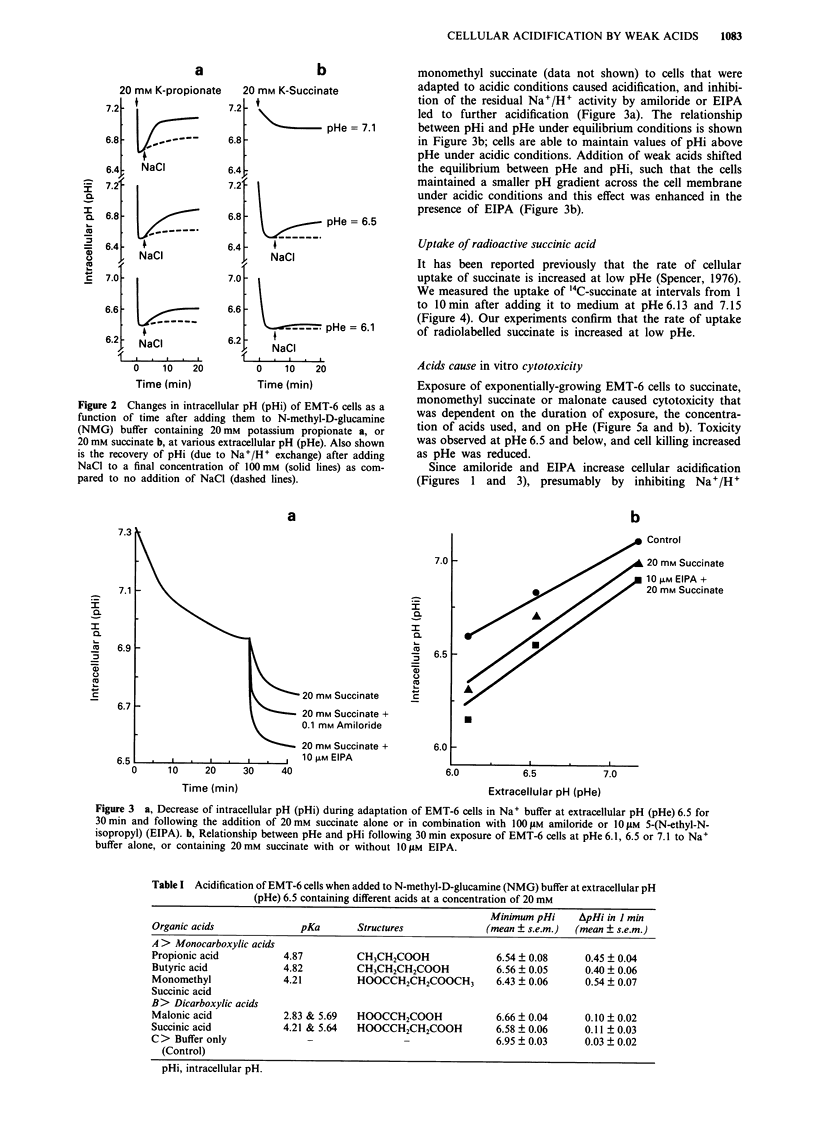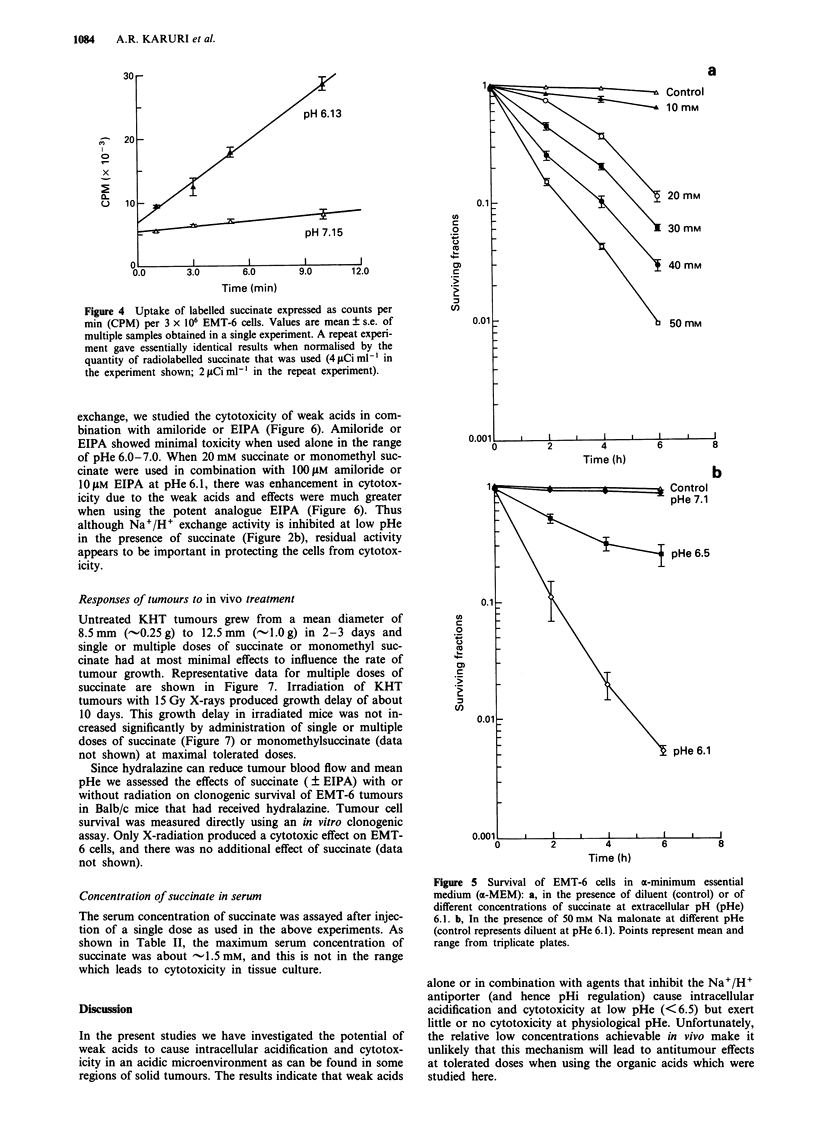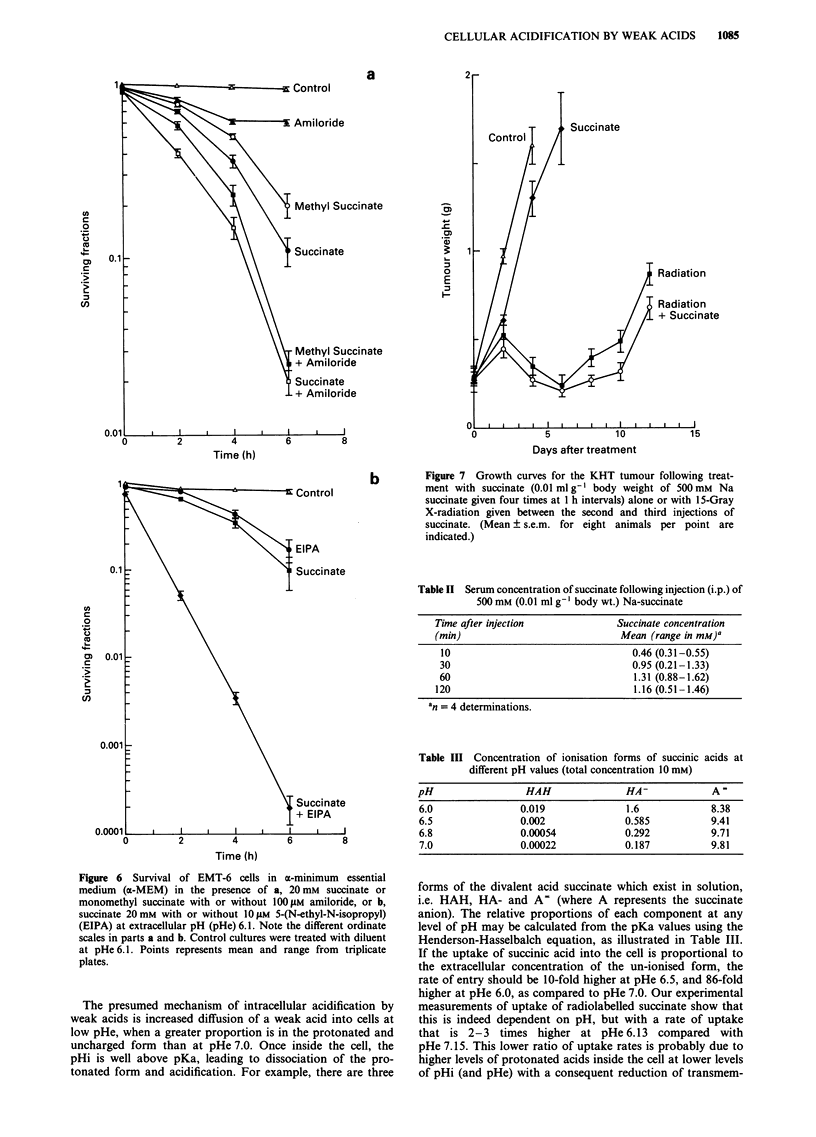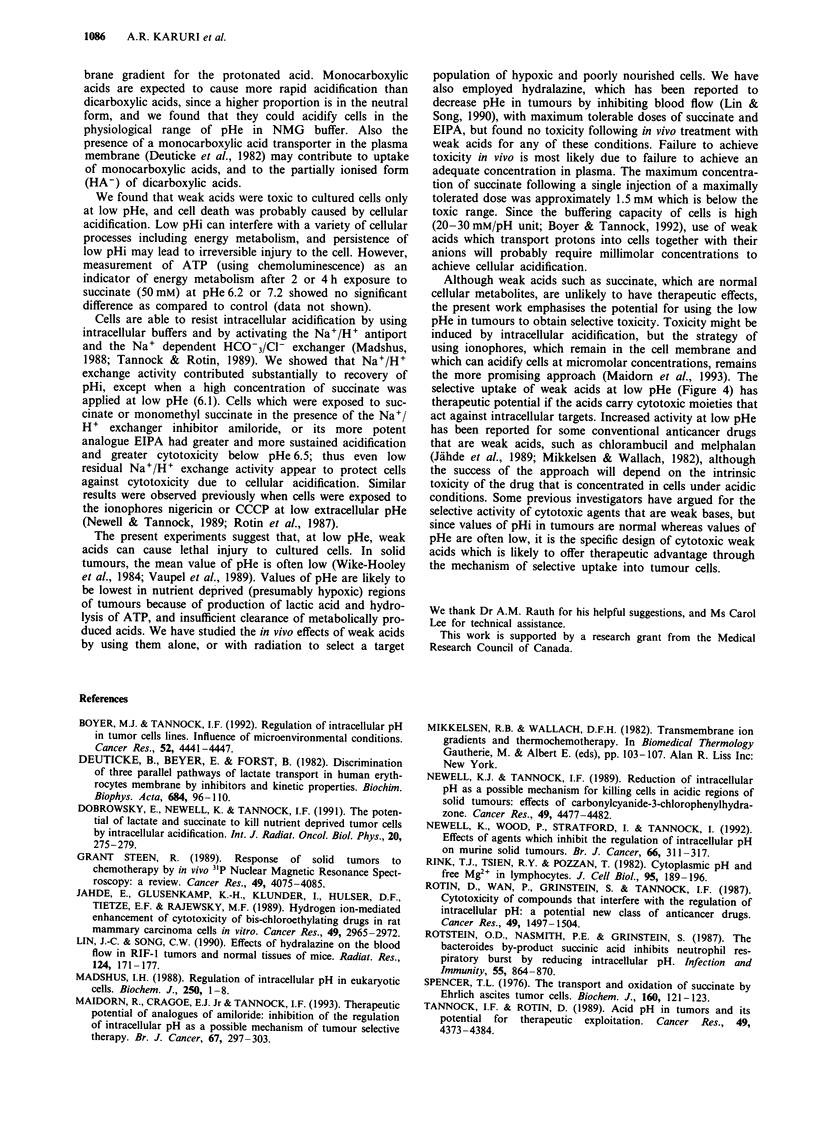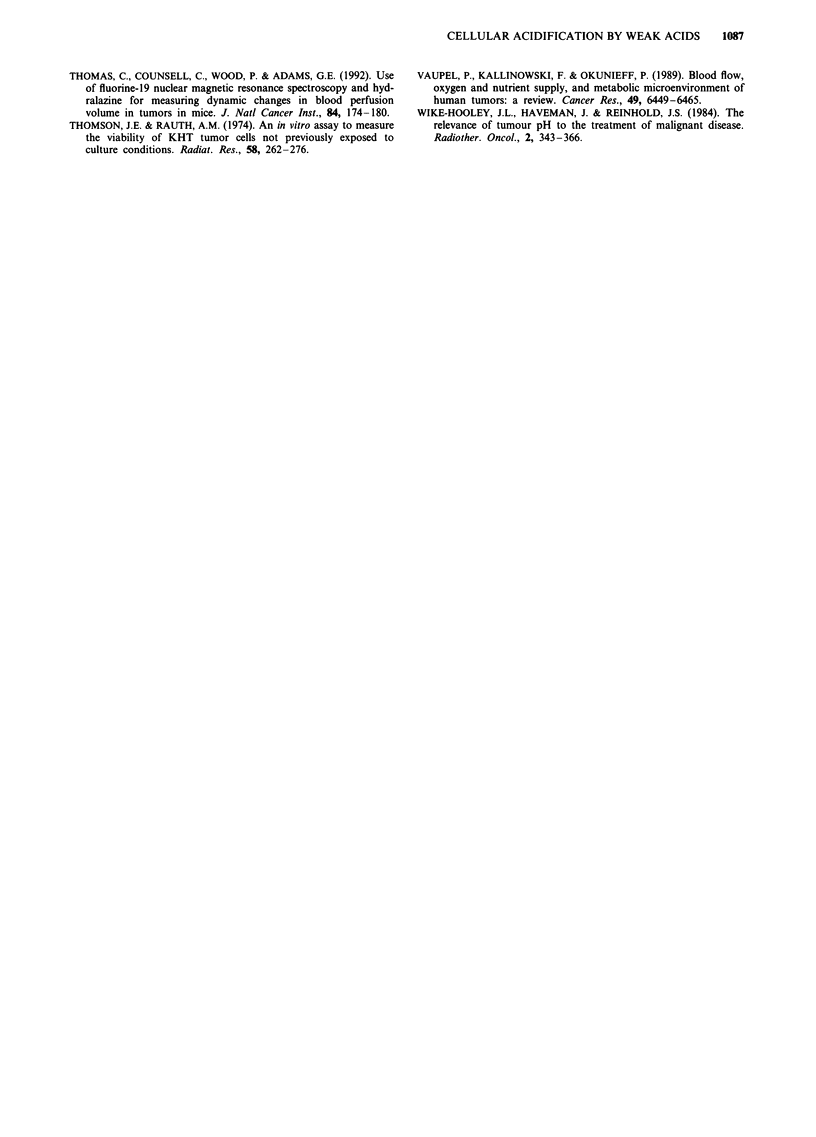# Selective cellular acidification and toxicity of weak organic acids in an acidic microenvironment.

**DOI:** 10.1038/bjc.1993.485

**Published:** 1993-12

**Authors:** A. R. Karuri, E. Dobrowsky, I. F. Tannock

**Affiliations:** Division of Experimental Therapeutics, Ontario Cancer Institute, Toronto, Canada.

## Abstract

The mean extracellular pH (pHe) within solid tumours has been found to be lower than in normal tissues. Agents which cause intracellular acidification at low pHe might have selective toxicity towards cells in tumours. Weak acids (or their anions) with pKa values in the range of 4-6 have a higher proportion of molecules in the uncharged form at low pHe and can diffuse more rapidly into cells. The effects of organic acids including succinate, monomethyl succinate and malonate to acidify cells have been evaluated under conditions of different pHe in the acidic range. These weak acids caused intracellular acidification of murine EMT-6 and human MGH-U1 cells in a concentration and pHe dependent fashion. At concentrations of 10 mM and above, these acids also caused in vitro cytotoxicity to these cells at low pHe (< 6.5). The rate and extent of cellular acidification caused by these weak acids, and their cytotoxicity at low pHe, were enhanced by exposure to amiloride and 5-(N-ethyl-N-isopropyl)amiloride (EIPA), agents which inhibit Na+/H+ exchange, and hence the regulation of intracellular pH. Acid dependent cytotoxicity was also investigated in a murine solid tumour using the endpoints of growth delay and colony formation in vitro following treatment in vivo. Agents were tested alone or with 15 Gy X-rays to select a population of hypoxic (and presumably acidic) cells. Achievable serum concentrations of succinate were about 1 mM and no antitumour activity of succinate was detected when used in this way. It is concluded that weak acids are selectively taken up into cells, and can cause selective cellular acidification and toxicity, at low pHe in culture. Weak acids that are normal cellular metabolites are not toxic in vivo, but weak acids carrying cytotoxic groups offer the potential for selective uptake and toxicity under the conditions of low pHe that exist in many solid tumours.


*Br. J. Cancer* (1993), **68**, 1080–1087 © Macmillan Press Ltd., 1993

### Selective cellular acidification and toxicity of weak organic acids in an acidic microenvironment

A.R. Karuri, E. Dobrowsky & I.F. Tannock

*Division of Experimental Therapeutics and Department of Medicine, Ontario Cancer Institute and University of Toronto, 500 Sherbourne Street, Toronto, Ontario, Canada M4X 1K9.*

**Summary** The mean extracellular pH (pHe) within solid tumours has been found to be lower than in normal tissues. Agents which cause intracellular acidification at low pHe might have selective toxicity towards cells in tumours. Weak acids (or their anions) with pKa values in the range of 4–6 have a higher proportion of molecules in the uncharged form at low pHe and can diffuse more rapidly into cells. The effects of organic acids including succinate, monomethyl succinate and malonate to acidify cells have been evaluated under conditions of different pHe in the acidic range. These weak acids caused intracellular acidification of murine EMT-6 and human MGH-U1 cells in a concentration and pHe dependent fashion. At concentrations of 10 mM and above, these acids also caused *in vitro* cytotoxicity to these cells at low pHe ($<6.5$). The rate and extent of cellular acidification caused by these weak acids, and their cytotoxicity at low pHe, were enhanced by exposure to amiloride and 5-(N-ethyl-N-isopropyl)amiloride (EIPA), agents which inhibit $Na^+/H^+$ exchange, and hence the regulation of intracellular pH. Acid dependent cytotoxicity was also investigated in a murine solid tumour using the endpoints of growth delay and colony formation *in vitro* following treatment *in vivo*. Agents were tested alone or with 15 Gy X-rays to select a population of hypoxic (and presumably acidic) cells. Achievable serum concentrations of succinate were about 1 mM and no antitumour activity of succinate was detected when used in this way. It is concluded that weak acids are selectively taken up into cells, and can cause selective cellular acidification and toxicity, at low pHe in culture. Weak acids that are normal cellular metabolites are not toxic *in vivo*, but weak acids carrying cytotoxic groups offer the potential for selective uptake and toxicity under the conditions of low pHe that exist in many solid tumours.

It has been shown that solid tumours of both humans and animals contain acidic regions (Vaupel *et al.*, 1989; Wike-Hooley *et al.*, 1984). The mean extracellular pH (pHe) within solid tumours has been shown from measurements with microelectrodes to be in the range of 6.5–7.0, which is about 0.5 units lower than in normal tissues. Extracellular acidity in tumours probably occurs because of relatively poor vascularisation as compared to normal tissues, leading to the poor clearance of acids produced by metabolism. Measurements of intracellular pH (pHi) in solid tumours made by nuclear magnetic resonance (NMR) spectroscopy have suggested that mean values of pHi are similar to those in normal tissues (Grant Steen, 1989; Vaupel *et al.*, 1989). Tumour cells are able to tolerate acidic pHe by using their buffering ability, and by activating ion exchange mechanisms which are present in the cell membrane, to maintain pHi close to the physiological range. Important membrane exchangers which regulate pHi in the acidic range are the $Na^+/H^+$ antiport and the $Na^+$ dependent $Cl^-/HCO^-_3$ exchanger (Madshus, 1988; Tannock & Rotin, 1989).

Previous studies in the present laboratory have shown that the ionophores nigericin and carbonylcyanide-3-chlorophenylhydrazone (CCCP) cause selective acidification of cells at low pHe; these agents are toxic at low but not at physiological pHe (Newell & Tannock, 1989; Rotin *et al.*, 1987) suggesting that toxicity is due to cellular acidification rather than to effects on mitochondria. The cytotoxicity of these agents was increased when they were used in combination with amiloride which inhibits the $Na^+/H^+$ antiporter, and with the stilbene derivative 4,4′-diisothiocyanostilbene 2,2-disulfonic acid (DIDS) which inhibits the $Na^+$-dependent $Cl^-/HCO^-_3$ exchanger.

Weak acids might also allow selective acidification of cells in an acidic environment. The protonated forms of weak acids are more membrane permeable than their charged anionic forms. Thus exposure of cells to weak acids is expected to result in an acute acid load to the cytoplasm which increases at lower pHe, where a higher proportion of acid is in the protonated and uncharged form. Preliminary evidence for acidification of tumour cells when exposed to the organic acid succinate was obtained previously (Dobrowsky *et al.*, 1991). Also, production of succinic acid by bacteroides, a group of anaerobic bacteria, has been shown to lead to a low pHe in abscesses and to cause intracellular acidification of granulocytes leading to loss of their viability (Rotstein *et al.*, 1988).

In the present paper we address the hypothesis that organic acids may cause acidification and death of cells at low pHe, and that these effects are increased by agents which inhibit regulation of pHi. We examine further whether such effects might allow selective treatment of tumour cells that are exposed to an acidic extracellular environment *in vivo*.

### Materials and methods

### Reagents

Succinic acid, malonic acid, succinic acid monomethyl ester, butyric acid, propionic acid, ATP-bioluminiscent assay kit and antimycin A were obtained from the Sigma Chemical Co., St. Louis, MO, USA. 2′,7′-bis(2-carboxyethyl)-5 (and 6-) carboxyfluorescein acetyomethyl ester (BCECF-AM) was purchased from Molecular Probes, Eugene, Oregon, USA. Radiolabelled 2,3-$^{14}$C-succinic acid (Sp. activity = 56 mCi $mmol^{-1}$) was obtained from New England Nuclear, Mississauga, Ontario, Canada.

### Cells

The murine mammary sarcoma cell line EMT-6 (obtained originally from Dr R. Sutherland, Rochester, NY, USA) and the human bladder carcinoma cell line MGH-U1 (obtained originally from the Urology Research Laboratory, Massachussetts General Hospital, Boston, MA, USA) were maintained in α-minimum essential medium (α-MEM) supplemented with 0.1 mg $ml^{-1}$ of kanamycin and 5% foetal bovine serum (FBS). These cell lines were chosen because their mechanisms of regulation of pHi have been studied in detail and because they allow comparison of effects against human and rodent cell lines (Boyer & Tannock, 1992). Both cell lines were reestablished from frozen stock at about 3

Correspondence: I.F. Tannock.
Received 9 March 1993; and in revised form 14 June 1993.

month intervals and were tested routinely for mycoplasma and were not contaminated. Cells were grown as monolayers in tissue culture flasks and were detached from flasks with 0.05% trypsin and 0.53 mM ethylenediaminetetraacetic acid (EDTA) (Gibco, Grand Island, NY, USA). All experiments were performed with exponentially growing cells.

*Measurement of intracellular pH*

Intracellular pH (pHi) was determined by using the pH sensitive fluorescent probe BCECF-AM (Rink *et al.*, 1982). Cells at a concentration of $1.5 \times 10^6$ ml$^{-1}$ in N-2-hydroxyethylpiperazine-N-ethanesulfonic acid (Hepes) buffered medium at pHe 7.4 were loaded with the esterified form of the fluorochrome, BCECF-AM, by incubation in the dark with 2 μg ml$^{-1}$ for 20 min at 37°C. Cells were washed by centrifugation and 80 μl of the cell suspension containing $\sim 5 \times 10^5$ cells was then added to a polystyrene cuvette containing NMG (140 mM N-Methyl-D-glucamine) or $Na^+$ (140 mM NaCl) each containing 1 mM KCl, 1 mM $CaCl_2$, 1 mM $MgCl_2$ and 5 mM glucose, buffered to different values of pHe with 20 mM Tris/Mes. The cuvette was temperature controlled (37°C) and stirred continuously using a magnetic flea. The uncharged form of BCECF-AM diffuses into the cell where it is cleaved by nonspecific esterases to form the charged, impermeant BCECF. Fluorescence was monitored with excitation and emission wavelengths set at 495 nm and 525 nm respectively. Weak acids (or their anionic forms) were titrated to desired values of pHe with KOH and were added to the cuvette in varying concentrations, and change in pHi was monitored with time. Calibration of pHi was performed after each experiment by adding 2 μg ml$^{-1}$ nigericin to a cuvette of BCECF-containing cells in $K^+$ buffer (identical to the Na buffer with isoosmotic replacement of KCl for NaCl). Nigericin sets pHi equal to pHe under these conditions and fluorescence was measured at varying pHe after sequential addition of aliquots of 1 M solutions of Mes[2-(N-morpholino)ethanesulfonic acid] (Mes) or Tris. This method of calibration has been supplemented in some experiments by lysing the cells and comparing fluorescence with measurements of pHe, with an appropriate correction for intracellular quenching of BCECF. There was an approximately linear relationship between pHi and fluorescence intensity in the range of pHi 6.0 to 7.5. The activity of the $Na^+/H^+$ exchanger was estimated from the rate of change of pHi following addition of 100 mM $Na^+$ to acid-loaded cells suspended in $Na^+$ free (NMG) solution (Madshus, 1988; Tannock & Rotin, 1989).

*Assessment of cytotoxicity* in vitro

Toxicity of weak acids at varying pHe was assessed by using the endpoint of colony formation. Five ml aliquots of a suspension containing $10^6$ cells ml$^{-1}$ in α-MEM plus FBS buffered to the required pH were added to small glass vials. The cells were stirred continuously at 37°C, and humidified air (plus 5% $CO_2$) flowed through the vials, as described previously (Rotin *et al.*, 1987). Weak acids were buffered to the same pH and added to the suspension 15 min later. At desired times after adding weak acids, 0.5 ml samples of the cell suspension were withdrawn by passing a long needle attached to a syringe through the gas outlet tube. The cells were washed and resuspended in α-MEM plus 5% FBS at pH 7.3, diluted, and plated in triplicate petri dishes. Colonies were stained with methylene blue and counted 9 to 13 days later. Surviving fraction was calculated as the ratio of plating efficiencies of treated and control plates exposed at the same pHe. Plating efficiency for control plates incubated at pHe 6.0 or above was always greater than 50%.

*Uptake of radiolabelled succinate*

Experiments involving uptake of radioactive succinate into EMT-6 cells were carried out in medium containing 140 mM NaCl, 1 mM KCl, 1 mM $MgCl_2$, 1 mM $CaCl_2$ which was buffered with Mes/Tris at 37°C to the desired pH; mixing was performed with a magnetic flea. Exponentially growing cells were trypsinised and washed with buffer solution at different pH without succinate. Cells were centrifuged and resuspended in isotonic solution at different pH containing 2 μM antimycin A to inhibit the oxidation of succinate (Spencer, 1976). After preincubation for 20 min, cells ($10^7$ ml$^{-1}$) were exposed to buffer at different pH which also contained 2 μM antimycin A, 5 mM unlabelled succinate and labelled succinate at a concentration 4 μCi ml$^{-1}$ (2 μCi ml$^{-1}$ in a repeat experiment). After different time periods, 0.3 ml aliquots were removed and nigericin was added to a final concentration of 5 μM; nigericin has been shown previously to cause almost complete inhibition of succinate transport (Spencer, 1976). Cells were centrifuged through a dibutylpthalate:corn oil (10:3) mixture to separate them from the buffer solution. Radioactivity was counted in the pellet using a liquid scintillation counter (LS 330; Beckman).

*Effects of weak acids on tumour growth*

Maximum tolerable doses of weak acids were determined in preliminary experiments by injecting different doses intraperitoneally into small groups of three mice. We determined the maximum dose at which animals survived with no abnormal behaviour, and with minimal weight loss. This dose was then used in subsequent experiments to study effects on growth of the KHT fibrosarcoma in syngeneic C3H/HeJ mice. This tumour is known to develop an acidic microenvironment with a mean value of pHe of $6.84 \pm 0.06$, and methods of transplantation and assessment of growth delay have been described previously (Newell *et al.*, 1992).

Appropriate dilutions of organic acids were titrated with NaOH to neutral pH and were injected intraperitoneally in volumes of 0.01 to 0.03 ml per gram body weight. Groups of mice bearing tumours with a mean diameter of 8.5 mm received either a single injection of succinate or monomethyl succinate or a course of four injections given at hourly intervals. To seek evidence for selective toxicity to hypoxic cells, a dose of 15 Gy was given immediately prior to a single injection, or between the second and third injections when multiple doses of the acid were given. Most cells which survive this radiation dose are expected to be hypoxic. Local tumour irradiation was delivered by using a specially designed 100 KV X-ray machine at a dose rate of 11.4 Gy min$^{-1}$ (Newell *et al.*, 1992). For assessment of the effect of treatment on tumour growth, tumour diameter was estimated on coded mice every second day by passing the tumour-bearing leg through a series of graded holes drilled in lucite. Mice were killed humanely when their tumours attained a mean diameter of 14 to 15 mm. Tumour growth curves were constructed from a previously defined calibration curve relating tumour weight to mean diameter.

*Effects of succinate on tumour cell survival* in vivo

We assessed clonogenic survival following treatment of the EMT-6 tumour, which is also known to develop an acidic microenvironment (mean pHe = $6.75 \pm 0.06$; Newell *et al.*, 1992). Balb/c mice weighing 20–24 g were injected with $5 \times 10^5$ EMT-6 cells into the hind flank. The tumours were allowed to grow for 7 days at which time the animals were randomised into groups to receive treatment. Treatments included four intraperitoneal injections of 0.01 ml g$^{-1}$ body weight of 500 mM Na-succinate or saline (controls) given at 1 h intervals; 15 Gy local X-irradiation given alone or between the second and third injections of Na-succinate; 5-(N-ethyl-N-isopropyl) amiloride (EIPA, 20 μM kg$^{-1}$ body weight) injected immediately after irradiation together with the same schedule of succinate injections; and hydralazine (10 mg kg$^{-1}$ body weight) injected with EIPA in a similar schedule. Hydralazine was given to try to inhibit tumour blood flow and thereby lower tumour pH (Lin & Song, 1990; Newell *et al.*, 1992; Thomas *et al.*, 1992). Tumours were removed from the mice 24 h after the last treatment and cell survival was estimated by using an *in vitro* clonogenic assay as described previously (Thomson & Rauth, 1974).

*Measurement of succinate in murine serum*

Succinate was assayed enzymatically by using a Boehringer Mannheim assay kit. Briefly, blood samples were collected from anaesthetised mice by puncturing the heart and serum was prepared from the blood by centrifugation. Measurement of the concentration of succinic acid in the serum utilises the following principle. Succinate is converted by the enzyme succinyl-CoA synthetase in the presence of inosine-5'-triphosphate (ITP) and Coenzyme A (CoA) to succinyl CoA with simultaneous formation of inosine-5'-diphosphate (IDP) and inorganic phosphate. Inosine-5'-diphosphate reacts with phosphoenol pyruvate in the presence of pyruvate kinase to pyruvate and ITP. Pyruvate is then reduced by NADH in the presence of lactate dehydrogenase (LDH). The amount of NADH oxidised in the above reaction is proportional to the amount of succinic acid originally present. Change in concentration of NADH was measured by its absorbance at 340 nm.

### Results

*Cellular acidification*

Experiments which characterise the effects of weak acids to cause intracellular acidification are described in Figures 1–3 and in Table I. All experiments were repeated to ensure reproducibility.

When mammalian cells grown at physiological pHe (~7.3) are added to medium buffered to a lower pHe there is a slow fall in pHi to a new equilibrium value; for EMT-6 cells at pHe 6.5 this equilibrium is about 6.9 (Table I and Figure 1). The monocarboxylic acids propionic acid, butyric acid and monomethyl succinic acid, and the dicarboxylic acids malonic acid and succinic acid all caused a marked increase in acidification of EMT-6 cells (Table I) and MGH-U1 cells (data not shown) at pHe 6.5. When cells, formerly grown at pHe 7.3 in α-MEM, were added to buffers containing these acids the rate of acidification was higher in NMG-containing buffer than in $Na^+$-containing buffer (Figures 1a and b) presumably because the presence of $Na^+$ allowed cellular acidification to be opposed by the activation of the $Na^+/H^+$ exchanger. Acidification of cells in the presence of $Na^+$ and the $Na^+/H^+$ inhibitor EIPA was more rapid than in $Na^+$-buffer, and only slightly slower than that observed in $Na^+$-free NMG containing buffer (Figure 1c).

The rate of acidification was dependent on concentration and on pHe for all acids used (Figures 1 and 2). The decrease in pHi was most rapid for cells exposed to monocarboxylic acids such as propionic acid, butyric acid and monomethyl succinic acid in NMG containing buffer (Table I) and these acids were able to acidify cells at neutral pHe 7.1; dicarboxylic acids did not cause cellular acidification at pHe 7.1 (Figure 2).

All mammalian cells express the $Na^+/H^+$ exchanger which allows exchange of extracellular $Na^+$ for intracellular $H^+$ under conditions when cells becomes acidified (Madshus, 1988; Tannock & Rotin, 1989). In order to measure $Na^+$-dependent pHi recovery from intracellular acidification by these weak acids, cells containing BCECF were exposed to different concentrations of weak acids in NMG-containing buffer at different pHe. When pHi had fallen to a low value, 100 mM NaCl was added, and $Na^+/H^+$ exchanger activity was measured by the rate of alkalinisation. Recoverability of pHi after acidification by these weak acids is concentration and pHe dependent but pHi returned to neutral in the presence of NaCl after addition of monocarboxylic acids at normal pHe (Figure 2). Exposure of cells to dicarboxylic acids (or their anions) such as succinate or malonate caused them to have slower recovery of pHi than exposure to monocarboxylic acids. At a concentration of 50 mM succinate there was almost complete inhibition of the $Na^+/H^+$ exchanger at pHe 6.1.

The above experiments have shown that organic acids increase the rate and extent of acidification when cells are placed acutely in medium at low pHe which contains them. However cells in an acidic microenvironment in tumours may be chronically adapted to low pHe. We have therefore performed additional experiments to determine whether organic acids were able to acidify cells that were adapted to low pHe.

When EMT-6 cells were exposed to $Na^+$ buffer at different pHe in the acidic range the cytoplasmic pHi equilibrated after about 30 min at a value which was dependent on (but higher than) pHe (Figure 3a). Addition of succinate or

**Figure 1** Changes in intracellular pH (pHi) of EMT-6 cells (previously grown in α-minimum essential medium (αMEM) at extracellular pH (pHe) 7.3) as a function of time after adding cells to N-methyl-D-glucamine (NMG) buffer **a**, or $Na^+$ buffer **b** and **c**, at pHe 6.5; the buffer contained double-distilled water (DDL) or different concentrations of succinate **a**–**c**, with or without 5-(N-ethyl-N-isopropyl) (EIPA, 10 μM; **c**).

**Figure 2** Changes in intracellular pH (pHi) of EMT-6 cells as a function of time after adding them to N-methyl-D-glucamine (NMG) buffer containing 20 mM potassium propionate **a**, or 20 mM succinate **b**, at various extracellular pH (pHe). Also shown is the recovery of pHi (due to $Na^+/H^+$ exchange) after adding NaCl to a final concentration of 100 mM (solid lines) as compared to no addition of NaCl (dashed lines).

monomethyl succinate (data not shown) to cells that were adapted to acidic conditions caused acidification, and inhibition of the residual $Na^+/H^+$ activity by amiloride or EIPA led to further acidification (Figure 3a). The relationship between pHi and pHe under equilibrium conditions is shown in Figure 3b; cells are able to maintain values of pHi above pHe under acidic conditions. Addition of weak acids shifted the equilibrium between pHe and pHi, such that the cells maintained a smaller pH gradient across the cell membrane under acidic conditions and this effect was enhanced in the presence of EIPA (Figure 3b).

### *Uptake of radioactive succinic acid*

It has been reported previously that the rate of cellular uptake of succinate is increased at low pHe (Spencer, 1976). We measured the uptake of $^{14}C$-succinate at intervals from 1 to 10 min after adding it to medium at pHe 6.13 and 7.15 (Figure 4). Our experiments confirm that the rate of uptake of radiolabelled succinate is increased at low pHe.

### *Acids cause* in vitro *cytotoxicity*

Exposure of exponentially-growing EMT-6 cells to succinate, monomethyl succinate or malonate caused cytotoxicity that was dependent on the duration of exposure, the concentration of acids used, and on pHe (Figure 5a and b). Toxicity was observed at pHe 6.5 and below, and cell killing increased as pHe was reduced.

Since amiloride and EIPA increase cellular acidification (Figures 1 and 3), presumably by inhibiting $Na^+/H^+$

**Figure 3** **a**, Decrease of intracellular pH (pHi) during adaptation of EMT-6 cells in $Na^+$ buffer at extracellular pH (pHe) 6.5 for 30 min and following the addition of 20 mM succinate alone or in combination with 100 µM amiloride or 10 µM 5-(N-ethyl-N-isopropyl) (EIPA). **b**, Relationship between pHe and pHi following 30 min exposure of EMT-6 cells at pHe 6.1, 6.5 or 7.1 to $Na^+$ buffer alone, or containing 20 mM succinate with or without 10 µM EIPA.

**Table I** Acidification of EMT-6 cells when added to N-methyl-D-glucamine (NMG) buffer at extracellular pH (pHe) 6.5 containing different acids at a concentration of 20 mM

| *Organic acids* | *pKa* | *Structures* | *Minimum pHi (mean ± s.e.m.)* | *ΔpHi in 1 min (mean ± s.e.m.)* |
|---|---|---|---|---|
| *A> Monocarboxylic acids* | | | | |
| Propionic acid | 4.87 | $CH_3CH_2COOH$ | 6.54 ± 0.08 | 0.45 ± 0.04 |
| Butyric acid | 4.82 | $CH_3CH_2CH_2COOH$ | 6.56 ± 0.05 | 0.40 ± 0.06 |
| Monomethyl Succinic acid | 4.21 | $HOOCCH_2CH_2COOCH_3$ | 6.43 ± 0.06 | 0.54 ± 0.07 |
| *B> Dicarboxylic acids* | | | | |
| Malonic acid | 2.83 & 5.69 | $HOOCCH_2COOH$ | 6.66 ± 0.04 | 0.10 ± 0.02 |
| Succinic acid | 4.21 & 5.64 | $HOOCCH_2CH_2COOH$ | 6.58 ± 0.06 | 0.11 ± 0.03 |
| C> Buffer only (Control) | – | – | 6.95 ± 0.03 | 0.03 ± 0.02 |

pHi, intracellular pH.

**Figure 4** Uptake of labelled succinate expressed as counts per min (CPM) per $3 \times 10^6$ EMT-6 cells. Values are mean ± s.e. of multiple samples obtained in a single experiment. A repeat experiment gave essentially identical results when normalised by the quantity of radiolabelled succinate that was used (4 $\mu$Ci $ml^{-1}$ in the experiment shown; 2 $\mu$Ci $ml^{-1}$ in the repeat experiment).

exchange, we studied the cytotoxicity of weak acids in combination with amiloride or EIPA (Figure 6). Amiloride or EIPA showed minimal toxicity when used alone in the range of pHe 6.0–7.0. When 20 mM succinate or monomethyl succinate were used in combination with 100 $\mu$M amiloride or 10 $\mu$M EIPA at pHe 6.1, there was enhancement in cytotoxicity due to the weak acids and effects were much greater when using the potent analogue EIPA (Figure 6). Thus although $Na^+/H^+$ exchange activity is inhibited at low pHe in the presence of succinate (Figure 2b), residual activity appears to be important in protecting the cells from cytotoxicity.

### *Responses of tumours to* in vivo *treatment*

Untreated KHT tumours grew from a mean diameter of 8.5 mm (~0.25 g) to 12.5 mm (~1.0 g) in 2–3 days and single or multiple doses of succinate or monomethyl succinate had at most minimal effects to influence the rate of tumour growth. Representative data for multiple doses of succinate are shown in Figure 7. Irradiation of KHT tumours with 15 Gy X-rays produced growth delay of about 10 days. This growth delay in irradiated mice was not increased significantly by administration of single or multiple doses of succinate (Figure 7) or monomethylsuccinate (data not shown) at maximal tolerated doses.

Since hydralazine can reduce tumour blood flow and mean pHe we assessed the effects of succinate (± EIPA) with or without radiation on clonogenic survival of EMT-6 tumours in Balb/c mice that had received hydralazine. Tumour cell survival was measured directly using an *in vitro* clonogenic assay. Only X-radiation produced a cytotoxic effect on EMT-6 cells, and there was no additional effect of succinate (data not shown).

### *Concentration of succinate in serum*

The serum concentration of succinate was assayed after injection of a single dose as used in the above experiments. As shown in Table II, the maximum serum concentration of succinate was about ~1.5 mM, and this is not in the range which leads to cytotoxicity in tissue culture.

### Discussion

In the present studies we have investigated the potential of weak acids to cause intracellular acidification and cytotoxicity in an acidic microenvironment as can be found in some regions of solid tumours. The results indicate that weak acids alone or in combination with agents that inhibit the $Na^+/H^+$ antiporter (and hence pHi regulation) cause intracellular acidification and cytotoxicity at low pHe (<6.5) but exert little or no cytotoxicity at physiological pHe. Unfortunately, the relative low concentrations achievable *in vivo* make it unlikely that this mechanism will lead to antitumour effects at tolerated doses when using the organic acids which were studied here.

**Figure 5** Survival of EMT-6 cells in α-minimum essential medium (α-MEM): **a**, in the presence of diluent (control) or of different concentrations of succinate at extracellular pH (pHe) 6.1. **b**, In the presence of 50 mM Na malonate at different pHe (control represents diluent at pHe 6.1). Points represent mean and range from triplicate plates.

**Figure 6** Survival of EMT-6 cells in α-minimum essential medium (α-MEM) in the presence of **a**, 20 mM succinate or monomethyl succinate with or without 100 μM amiloride, or **b**, succinate 20 mM with or without 10 μM 5-(N-ethyl-N-isopropyl) (EIPA) at extracellular pH (pHe) 6.1. Note the different ordinate scales in parts **a** and **b**. Control cultures were treated with diluent at pHe 6.1. Points represents mean and range from triplicate plates.

The presumed mechanism of intracellular acidification by weak acids is increased diffusion of a weak acid into cells at low pHe, when a greater proportion is in the protonated and uncharged form than at pHe 7.0. Once inside the cell, the pHi is well above pKa, leading to dissociation of the protonated form and acidification. For example, there are three

**Figure 7** Growth curves for the KHT tumour following treatment with succinate (0.01 ml $g^{-1}$ body weight of 500 mM Na succinate given four times at 1 h intervals) alone or with 15-Gray X-radiation given between the second and third injections of succinate. (Mean ± s.e.m. for eight animals per point are indicated.)

**Table II** Serum concentration of succinate following injection (i.p.) of 500 mM (0.01 ml $g^{-1}$ body wt.) Na-succinate

| *Time after injection (min)* | *Succinate concentration Mean (range in mM)*[a] |
|---|---|
| 10 | 0.46 (0.31–0.55) |
| 30 | 0.95 (0.21–1.33) |
| 60 | 1.31 (0.88–1.62) |
| 120 | 1.16 (0.51–1.46) |

[a]*n* = 4 determinations.

**Table III** Concentration of ionisation forms of succinic acids at different pH values (total concentration 10 mM)

| *pH* | *HAH* | *$HA^-$* | $A^=$ |
|---|---|---|---|
| 6.0 | 0.019 | 1.6 | 8.38 |
| 6.5 | 0.002 | 0.585 | 9.41 |
| 6.8 | 0.00054 | 0.292 | 9.71 |
| 7.0 | 0.00022 | 0.187 | 9.81 |

forms of the divalent acid succinate which exist in solution, i.e. HAH, HA- and $A^=$ (where A represents the succinate anion). The relative proportions of each component at any level of pH may be calculated from the pKa values using the Henderson-Hasselbalch equation, as illustrated in Table III. If the uptake of succinic acid into the cell is proportional to the extracellular concentration of the un-ionised form, the rate of entry should be 10-fold higher at pHe 6.5, and 86-fold higher at pHe 6.0, as compared to pHe 7.0. Our experimental measurements of uptake of radiolabelled succinate show that this is indeed dependent on pH, but with a rate of uptake that is 2–3 times higher at pHe 6.13 compared with pHe 7.15. This lower ratio of uptake rates is probably due to higher levels of protonated acids inside the cell at lower levels of pHi (and pHe) with a consequent reduction of transmembrane gradient for the protonated acid. Monocarboxylic acids are expected to cause more rapid acidification than dicarboxylic acids, since a higher proportion is in the neutral form, and we found that they could acidify cells in the physiological range of pHe in NMG buffer. Also the presence of a monocarboxylic acid transporter in the plasma membrane (Deuticke *et al.*, 1982) may contribute to uptake of monocarboxylic acids, and to the partially ionised form ($HA^-$) of dicarboxylic acids.

We found that weak acids were toxic to cultured cells only at low pHe, and cell death was probably caused by cellular acidification. Low pHi can interfere with a variety of cellular processes including energy metabolism, and persistence of low pHi may lead to irreversible injury to the cell. However, measurement of ATP (using chemoluminescence) as an indicator of energy metabolism after 2 or 4 h exposure to succinate (50 mM) at pHe 6.2 or 7.2 showed no significant difference as compared to control (data not shown).

Cells are able to resist intracellular acidification by using intracellular buffers and by activating the $Na^+/H^+$ antiport and the $Na^+$ dependent $HCO^-{}_3/Cl^-$ exchanger (Madshus, 1988; Tannock & Rotin, 1989). We showed that $Na^+/H^+$ exchange activity contributed substantially to recovery of pHi, except when a high concentration of succinate was applied at low pHe (6.1). Cells which were exposed to succinate or monomethyl succinate in the presence of the $Na^+/H^+$ exchanger inhibitor amiloride, or its more potent analogue EIPA had greater and more sustained acidification and greater cytotoxicity below pHe 6.5; thus even low residual $Na^+/H^+$ exchange activity appear to protect cells against cytotoxicity due to cellular acidification. Similar results were observed previously when cells were exposed to the ionophores nigericin or CCCP at low extracellular pHe (Newell & Tannock, 1989; Rotin *et al.*, 1987).

The present experiments suggest that, at low pHe, weak acids can cause lethal injury to cultured cells. In solid tumours, the mean value of pHe is often low (Wike-Hooley *et al.*, 1984; Vaupel *et al.*, 1989). Values of pHe are likely to be lowest in nutrient deprived (presumably hypoxic) regions of tumours because of production of lactic acid and hydrolysis of ATP, and insufficient clearance of metabolically produced acids. We have studied the *in vivo* effects of weak acids by using them alone, or with radiation to select a target population of hypoxic and poorly nourished cells. We have also employed hydralazine, which has been reported to decrease pHe in tumours by inhibiting blood flow (Lin & Song, 1990), with maximum tolerable doses of succinate and EIPA, but found no toxicity following *in vivo* treatment with weak acids for any of these conditions. Failure to achieve toxicity *in vivo* is most likely due to failure to achieve an adequate concentration in plasma. The maximum concentration of succinate following a single injection of a maximally tolerated dose was approximately 1.5 mM which is below the toxic range. Since the buffering capacity of cells is high (20–30 mM/pH unit; Boyer & Tannock, 1992), use of weak acids which transport protons into cells together with their anions will probably require millimolar concentrations to achieve cellular acidification.

Although weak acids such as succinate, which are normal cellular metabolites, are unlikely to have therapeutic effects, the present work emphasises the potential for using the low pHe in tumours to obtain selective toxicity. Toxicity might be induced by intracellular acidification, but the strategy of using ionophores, which remain in the cell membrane and which can acidify cells at micromolar concentrations, remains the more promising approach (Maidorn *et al.*, 1993). The selective uptake of weak acids at low pHe (Figure 4) has therapeutic potential if the acids carry cytotoxic moieties that act against intracellular targets. Increased activity at low pHe has been reported for some conventional anticancer drugs that are weak acids, such as chlorambucil and melphalan (Jähde *et al.*, 1989; Mikkelsen & Wallach, 1982), although the success of the approach will depend on the intrinsic toxicity of the drug that is concentrated in cells under acidic conditions. Some previous investigators have argued for the selective activity of cytotoxic agents that are weak bases, but since values of pHi in tumours are normal whereas values of pHe are often low, it is the specific design of cytotoxic weak acids which is likely to offer therapeutic advantage through the mechanism of selective uptake into tumour cells.

We thank Dr A.M. Rauth for his helpful suggestions, and Ms Carol Lee for technical assistance.

This work is supported by a research grant from the Medical Research Council of Canada.

### References

BOYER, M.J. & TANNOCK, I.F. (1992). Regulation of intracellular pH in tumor cells lines. Influence of microenvironmental conditions. *Cancer Res.*, **52**, 4441–4447.

DEUTICKE, B., BEYER, E. & FORST, B. (1982). Discrimination of three parallel pathways of lactate transport in human erythrocytes membrane by inhibitors and kinetic properties. *Biochim. Biophys. Acta*, **684**, 96–110.

DOBROWSKY, E., NEWELL, K. & TANNOCK, I.F. (1991). The potential of lactate and succinate to kill nutrient deprived tumor cells by intracellular acidification. *Int. J. Radiat. Oncol. Biol. Phys.*, **20**, 275–279.

GRANT STEEN, R. (1989). Response of solid tumors to chemotherapy by *in vivo* $^{31}P$ Nuclear Magnetic Resonance Spectroscopy: a review. *Cancer Res.*, **49**, 4075–4085.

JAHDE, E., GLUSENKAMP, K.-H., KLUNDER, I., HULSER, D.F., TIETZE, E.F. & RAJEWSKY, M.F. (1989). Hydrogen ion-mediated enhancement of cytotoxicity of bis-chloroethylating drugs in rat mammary carcinoma cells *in vitro*. *Cancer Res.*, **49**, 2965–2972.

LIN, J.-C. & SONG, C.W. (1990). Effects of hydralazine on the blood flow in RIF-1 tumors and normal tissues of mice. *Radiat. Res.*, **124**, 171–177.

MADSHUS, I.H. (1988). Regulation of intracellular pH in eukaryotic cells. *Biochem. J.*, **250**, 1–8.

MAIDORN, R., CRAGOE, E.J. Jr & TANNOCK, I.F. (1993). Therapeutic potential of analogues of amiloride: inhibition of the regulation of intracellular pH as a possible mechanism of tumour selective therapy. *Br. J. Cancer*, **67**, 297–303.

MIKKELSEN, R.B. & WALLACH, D.F.H. (1982). Transmembrane ion gradients and thermochemotherapy. In *Biomedical Thermology* Gautherie, M. & Albert E. (eds), pp. 103–107. Alan R. Liss Inc: New York.

NEWELL, K.J. & TANNOCK, I.F. (1989). Reduction of intracellular pH as a possible mechanism for killing cells in acidic regions of solid tumours: effects of carbonylcyanide-3-chlorophenylhydrazone. *Cancer Res.*, **49**, 4477–4482.

NEWELL, K., WOOD, P., STRATFORD, I. & TANNOCK, I. (1992). Effects of agents which inhibit the regulation of intracellular pH on murine solid tumours. *Br. J. Cancer*, **66**, 311–317.

RINK, T.J., TSIEN, R.Y. & POZZAN, T. (1982). Cytoplasmic pH and free $Mg^{2+}$ in lymphocytes. *J. Cell Biol.*, **95**, 189–196.

ROTIN, D., WAN, P., GRINSTEIN, S. & TANNOCK, I.F. (1987). Cytotoxicity of compounds that interfere with the regulation of intracellular pH: a potential new class of anticancer drugs. *Cancer Res.*, **49**, 1497–1504.

ROTSTEIN, O.D., NASMITH, P.E. & GRINSTEIN, S. (1987). The bacteroides by-product succinic acid inhibits neutrophil respiratory burst by reducing intracellular pH. *Infection and Immunity*, **55**, 864–870.

SPENCER, T.L. (1976). The transport and oxidation of succinate by Ehrlich ascites tumor cells. *Biochem. J.*, **160**, 121–123.

TANNOCK, I.F. & ROTIN, D. (1989). Acid pH in tumors and its potential for therapeutic exploitation. *Cancer Res.*, **49**, 4373–4384.

THOMAS, C., COUNSELL, C., WOOD, P. & ADAMS, G.E. (1992). Use of fluorine-19 nuclear magnetic resonance spectroscopy and hydralazine for measuring dynamic changes in blood perfusion volume in tumors in mice. *J. Natl Cancer Inst.*, **84**, 174–180.

THOMSON, J.E. & RAUTH, A.M. (1974). An *in vitro* assay to measure the viability of KHT tumor cells not previously exposed to culture conditions. *Radiat. Res.*, **58**, 262–276.

VAUPEL, P., KALLINOWSKI, F. & OKUNIEFF, P. (1989). Blood flow, oxygen and nutrient supply, and metabolic microenvironment of human tumors: a review. *Cancer Res.*, **49**, 6449–6465.

WIKE-HOOLEY, J.L., HAVEMAN, J. & REINHOLD, J.S. (1984). The relevance of tumour pH to the treatment of malignant disease. *Radiother. Oncol.*, **2**, 343–366.